# Cuproptosis in spinal cord injury: emerging mechanisms and immunological relevance

**DOI:** 10.1080/07853890.2026.2641867

**Published:** 2026-03-25

**Authors:** Lu-Hao Li, Jie-qi Zhang, Xi-han Ying, Yi Huang, Cheng-cheng Xu, Xiao-xiang Wang, Ke-lin He, Rui-jie Ma

**Affiliations:** a The Third School of Clinical Medicine (School of Rehabilitation Medicine), Zhejiang Chinese Medical University, Hangzhou, China; b Key Laboratory of Acupuncture and Neurology of Zhejiang Province, The Third School of Clinical Medicine (School of Rehabilitation Medicine), Zhejiang Chinese Medical University, Hangzhou, China; c Department of Acupuncture, The Third Affiliated Hospital of Zhejiang Chinese Medical University (Zhongshan Hospital of Zhejiang Province), Hangzhou, China

**Keywords:** Spinal cord injury, cuproptosis, immune microenvironment

## Abstract

**Background:**

Spinal cord injury (SCI) is a severe neurological disorder involving a two-phase pathological process. The primary injury results from direct mechanical trauma, while the secondary injury includes a cascade of cellular and molecular events such as excitotoxicity, oxidative stress, and inflammation. Recent studies have highlighted cuproptosis, a novel form of copper-dependent regulated cell death, as a significant contributor to secondary injury and immune dysregulation.

**Objective:**

To summarize current research on cuproptosis in the context of spinal cord injury, clarify its molecular mechanisms and immunological effects, and explore its potential as a therapeutic target.

**Methods:**

Relevant experimental and review studies were identified through database searches using keywords including cuproptosis, spinal cord injury, copper homeostasis, and regulated cell death. The review focuses on molecular mechanisms, mitochondrial dysfunction, immune responses, and key regulators such as FDX1, DLAT, ATP7A, and CTR1.

**Results:**

Following spinal cord injury, disruption of the blood–spinal cord barrier and inflammatory responses lead to copper accumulation, triggering cuproptosis. This process causes protein aggregation, mitochondrial dysfunction, and cell death, particularly in neurons, oligodendrocytes, and activated immune cells. During the acute phase, cuproptosis intensifies neuronal loss and inflammation. In the chronic phase, it may help eliminate persistently activated cells and contribute to scar remodeling. Cuproptosis also interacts with other forms of regulated cell death, such as pyroptosis and ferroptosis.

**Conclusion:**

Cuproptosis plays an important role in SCI secondary injury and immune microenvironment remodeling. Targeting cuproptosis related pathways and restoring copper homeostasis may provide promising strategies for precision therapy.

## 1. Introduction

Spinal cord injury (SCI) is a severe traumatic disease of the central nervous system, often leading to long-term or even lifelong impairments in motor, sensory, and autonomic functions [1]. Its pathological process typically includes two stages: primary injury and secondary injury. The primary injury is caused by acute mechanical damage to neurons, glial cells, and vascular structures due to transient external forces, which is irreversible. Secondary injury, on the other hand, progressively expands from hours to weeks after the initial damage, and is a crucial determinant of the extent of tissue necrosis and functional outcomes [1]. This stage is centered around mitochondrial dysfunction and metabolic disorders, accompanied by excitotoxicity, oxidative stress, inflammation amplification, and the overlapping and synergistic effects of various regulated cell death processes, ultimately leading to continued loss of neurons and oligodendrocytes, demyelination of white matter, and glial scar formation, thereby significantly limiting the recovery of neural function [1].

In recent years, the disruption of metal ion homeostasis has been recognized as an important upstream factor in secondary injury following SCI, with particular attention given to copper dyshomeostasis and elevated copper levels in the injured microenvironment [2]. Copper is an essential trace element in the human body, involved in various physiological processes, including the regulation of redox reactions, energy metabolism, and the normal function of several metalloenzymes [3]. However, free copper is highly chemically active, and its excessive accumulation can exert toxic effects on cells by promoting harmful oxidative reactions and disrupting protein structural stability [3].

Under normal physiological conditions, the body maintains copper homeostasis through a complex and finely regulated system, including copper uptake, distribution within cells, buffering, and excretion [3]. These processes work together to effectively limit the level of reactive free copper. After SCI, factors such as the disruption of the blood-spinal cord barrier, local hemorrhage, persistent inflammation, and oxidative stress can disrupt these regulatory processes, leading to an increase in the level of exchangeable free copper in the tissue microenvironment [4,5]. This change can not only directly damage the structure and function of mitochondria but may also exacerbate secondary injury through enhanced oxidative stress and inflammation [4].

In this context, cuproptosis, a recently proposed form of regulated cell death, provides a mechanistic framework for understanding the link between copper toxicity and mitochondrial metabolic dysfunction. Existing research suggests that cuproptosis is closely related to mitochondrial metabolic status, especially depending on the acetylation modification of proteins involved in the tricarboxylic acid cycle. One of its key molecules, ferredoxin 1 (FDX1), promotes copper ions to be in a state that is more prone to participate in reactions, enhancing the interaction between copper and acetylated proteins. When copper load increases, copper ions can bind to acetylated proteins in the tricarboxylic acid cycle (such as DLAT) and induce abnormal aggregation, which may also lead to a decline in the stability of iron-sulfur cluster proteins. These changes cause significant protein toxicity stress and impair mitochondrial respiratory chain function, ultimately leading to energy metabolic collapse and cell death. Unlike ferroptosis, which is primarily driven by iron-dependent lipid peroxidation and an imbalance in the GPX4 antioxidant system, cuproptosis is more prominently characterized by the aggregation of acetylated metabolic proteins and damage to iron-sulfur cluster proteins, resulting in a mitochondrial protein homeostasis crisis. In contrast to pyroptosis, which is characterized by the activation of inflammasomes and membrane pore formation mediated by gasdermin, the core change in cuproptosis does not primarily manifest as membrane pore formation but originates from the imbalance in the structure and function of mitochondrial metabolic proteins [3]. Current research on cuproptosis in SCI is still in its early stages, but studies suggest that dysregulation of copper homeostasis after SCI may increase the susceptibility of neurons and glial cells to this process and may influence the immune microenvironment remodeling after injury through mitochondrial stress-related signaling and the release of injury-related molecules [6]. Based on this, this paper will systematically review the research progress of cuproptosis in SCI, focusing on its molecular mechanisms, cell type differences, and potential immunological significance, and discuss related intervention strategies and translational research directions, with the aim of providing a clearer theoretical basis for subsequent mechanism verification and clinical exploration.

## 2. Pathological mechanisms and immunological features of spinal cord injury

### 2.1. Primary injury mechanism

Primary spinal cord injury (SCI) is caused by a sudden mechanical force, which can cause direct damage to the spinal cord parenchyma and vascular system in a very short period of time. This is manifested by axonal transection, intramedullary hemorrhage, and acute deformation and damage of gray and white matter structures [7]. Following this, microcirculatory perfusion impairment promotes the rapid adhesion of peripheral white blood cells to the endothelial surface of blood vessels, which subsequently migrate across the endothelium into the spinal cord tissue [4]. These events together lead to a significant increase in the permeability of the blood–spinal cord barrier, allowing plasma components and peripheral immune cells to infiltrate the spinal cord parenchyma and the surrounding damaged area, thereby participating in the local inflammatory response and the progression of pathological processes in the early stages [4].

At the cellular level, the increase in membrane permeability and excitotoxicity signals quickly disrupt ion homeostasis; calcium overload and glutamate-mediated excitotoxicity can lead to mitochondrial depolarization, ATP depletion, and promote early necrosis or apoptotic cell death in neurons and oligodendrocytes [8]. Meanwhile, damaged cells release damage-associated molecular patterns (DAMPs), including HMGB1, which triggers the innate immune response and drives the formation of secondary inflammatory processes [9].

The bleeding and barrier dysfunction induced by the primary injury may further alter the local metabolic environment and reduce the mitochondrial tolerance to stress, thus laying the foundation for the subsequent metabolic vulnerability [10].

### 2.2. Secondary injury mechanisms

#### 2.2.1. Oxidative stress

Oxidative stress is a major driving factor in the secondary pathological progression of SCI, and its occurrence is closely related to ischemia-reperfusion, excitotoxicity, ion homeostasis disturbances, and inflammation-related metabolic load [11,12]. Mitochondria are both a significant source of reactive oxygen species (ROS) and reactive nitrogen species (RNS), and under prolonged stress, they become the primary targets of damage [13]. In parallel with antioxidant and metabolic interventions, emerging approaches such as mitochondrial transfer or transplantation have been reviewed as potential strategies to restore bioenergetics and mitigate secondary injury cascades after SCI [14]. After injury, intracellular calcium levels rise, accompanied by dysfunction of the electron transport chain, which predisposes to the abnormal accumulation of reactive species, leading to a decline in mitochondrial membrane potential, reduced oxidative phosphorylation efficiency, and gradual bioenergy failure [15]. These changes not only directly damage proteins, lipids, and nucleic acids but may also produce diffusible toxic products through lipid peroxidation, spreading the damage effect to surrounding tissues and further amplifying local inflammatory signals [15].

When mitochondrial function is impaired, leading to insufficient NADPH supply and limited glutathione cycle regeneration, endogenous cellular antioxidant defenses often fail to adequately counteract persistent oxidative stress [16]. Even when the Nrf2-related protective mechanism is activated, it may be unable to maintain stable antioxidant capacity [17].

It is important to note that oxidative stress is not an isolated process. It may interact with regulated cell death mechanisms, such as ferroptosis, which in turn influences the sensitivity of different cell types to metal-related metabolic stress, and to some extent, regulates the tissue’s tolerance threshold to damage and the direction of the pathological progression [18].

#### 2.2.2. Inflammatory response

The inflammatory response is another key component of secondary injury following SCI, typically initiated by the release of DAMPs and the disruption of the blood-spinal cord barrier (BSCB). It is gradually amplified through the activation of resident microglia and astrocytes, along with the recruitment of peripheral immune cells [19]. The release of inflammatory mediators and oxidative bursts can further exacerbate mitochondrial dysfunction, promoting demyelination and neuronal loss [20]. If the inflammatory response is not resolved in a timely manner, it may also promote scar formation and tissue structural remodeling, ultimately leading to long-term functional impairments [20].

During the secondary injury phase, the inflammatory response not only directly triggers the accumulation of cytokines and oxidative bursts but also depletes the buffering capacity of the antioxidant system, interferes with the processing of metal ions, and other mechanisms, making the injured area more prone to a sustained state of metabolic and inflammatory imbalance [21,22].

It is important to emphasize that the activation state of immune cells is closely related to their metabolic pathways. Pro-inflammatory myeloid cells tend to rely on enhanced glycolysis, while phenotypes that promote tissue repair are more dependent on mitochondrial oxidative phosphorylation and fatty acid metabolism [23]. Based on these metabolic preferences, mitochondrial dysfunction caused by metal exposure, along with associated oxidative stress and energy metabolic pressures, can induce varying degrees of metabolic adjustments and functional changes in myeloid cells with different activation states. This, in turn, influences whether the inflammatory response remains persistent or gradually progresses toward repair and homeostasis restoration [24].

Moreover, adaptive immune cells are equally sensitive to the metabolic changes induced by injury [25]. Since lymphocyte activation and immune regulatory functions largely depend on the support of mitochondrial metabolism, metal-related stress and mitochondrial dysfunction may indirectly disrupt the balance between pro-inflammatory and immune regulation, thereby contributing to shaping the post-injury immune microenvironment [26,27].

### 2.3. Immune response in spinal cord injury

#### 2.3.1. Acute immune response after SCI

The acute immune response following SCI typically begins within hours. Necrotic cell debris and DAMPs rapidly activate the innate immune system and, in the context of a disrupted blood-spinal cord barrier (BSCB), promote leukocyte infiltration [28]. Early infiltrating neutrophils and monocyte-derived macrophages, together with activated microglia, jointly dominate the early immune environment at the lesion site. They influence the survival of neurons and glial cells by releasing reactive substances and various cytokines [29].

On a systemic level, acute SCI can induce a significant imbalance in neuro-immune regulation, with peripheral immune responses exhibiting a complex pattern of both inflammatory activation and immune suppression. This can manifest as SCI-related immune dysfunction syndrome (SCI-IDS)[30]. In clinical populations, immune phenotypic changes associated with immune suppression, such as a decrease in monocyte antigen presentation markers (e.g. mHLA-DR/MHC II), are observed following acute SCI, and this change is correlated with an increased risk of infections [30]. Such immune alterations are typically closely related to the severity of the injury and may persist for several weeks [30].

During the acute phase of SCI, immune cells are generally under dual challenges of high metabolic load and significant oxidative stress. Their functional state is sensitive to fluctuations in the local ­metabolic environment, and this sensitivity may further influence their immune effects and the response patterns to the injured area [11].

#### 2.3.2. Chronic immune response and repair after SCI

In the chronic phase of SCI, the local immune environment gradually shifts to a state of persistent low-grade inflammation, accompanied by scar-related remodeling and long-term changes in the function of resident glial cells [21]. Microglia and macrophages continue to play a central role in the immune response following injury, but they exhibit significant heterogeneity in their functional states and phenotypes. Some subpopulations may remain in an activated state for extended periods, continuously releasing pro-inflammatory factors. This sustained inflammatory response can interfere with the restoration of local homeostasis, thereby hindering effective tissue repair [31].

Astrocytes and other extracellular matrix components work together to form the injury boundary and participate in extracellular matrix remodeling, which, to some extent, helps limit lesion expansion. However, this process also creates an inhibitory environment that is unfavorable for axonal regeneration and remyelination [21]. The continuous loss of oligodendrocytes and the limited differentiation of oligodendrocyte precursor cells can lead to chronic demyelination, while the prolonged presence of immune signaling and oxidative stress further restricts myelin recovery [32]. Adaptive immune changes may also persist, promoting the maintenance of chronic inflammation and tissue remodeling [21]. Beyond astroglial scarring, fibrotic scarring is increasingly recognized as a mechanosensitive barrier to regeneration. A recent study reported that tuning hydrogel mechanical strength to match spinal cord tissue can guide fibroblast alignment and remodel extracellular matrix (ECM) deposition into a more permissive ‘parallel matrix’. The study further identified an Il11ra1^+^/Itga11^+^ fibroblast subpopulation associated with aligned infiltration and suggested that it may promote axonal growth at the lesion site through an LRP6/β-catenin/MMP7 mechanotransduction cascade [33]. In addition, chronic-stage alterations after SCI are not confined to the lesion microenvironment. In a recent polytrauma animal model incorporating SCI, SCI-associated heterotopic ossification (HO) could be recapitulated, suggesting that long-term inflammatory–mesenchymal dysregulation and systemic remodeling may contribute to late pathological evolution [34]. Collectively, persistent local inflammation and scar-related barriers, together with potential systemic remodeling, jointly constrain reparative capacity and exacerbate regenerative failure in the chronic phase.

## 3. Molecular mechanisms underlying cuproptosis

### 3.1. Copper homeostasis and cuproptosis in spinal cord injury

As a vital micronutrient, copper is required to sustain normal physiological functions and participates in key biological processes such as energy production, redox regulation, cellular structural integrity, and metal ion homeostasis [35]. Its biological functions primarily depend on serving as a cofactor for various metalloenzymes. For instance, cytochrome c oxidase (CCO) regulates oxidative phosphorylation to facilitate adenosine triphosphate (ATP) generation; copper/zinc superoxide dismutase (Cu/Zn-SOD) eliminates excess ROS to preserve cellular redox homeostasis; lysyl oxidase (LOX) participates in extracellular matrix crosslinking; and ceruloplasmin (CP) is critically involved in iron metabolism and transport [36–38]. However, copper ions possess extremely high redox activity, and when present in a free state, they can readily trigger cytotoxic reactions; therefore, intracellular copper levels must be strictly regulated [39].

During dietary absorption, cupric ions (Cu^2+^) must first be reduced to cuprous ions (Cu^+^) by six-transmembrane epithelial antigen of the prostate (STEAP) proteins [40]. The reduced Cu^+^ is subsequently transported into intestinal epithelial cells primarily *via* copper transporter 1 (CTR1, also known as solute carrier family 31 member 1, SLC31A1), while divalent metal transporter 1 (DMT1, also known as solute carrier family 11 member 2, SLC11A2) can also serve as an auxiliary pathway for copper uptake [40]. Once inside the cell, copper is distributed through specific metallochaperones, such as antioxidant protein 1 (ATOX1), copper chaperone for superoxide dismutase (CCS), and cytochrome c oxidase copper chaperone (COX17), which mediate its targeted delivery. Specifically, CCS transfers copper to copper/zinc superoxide dismutase 1 (Cu/Zn-SOD1), enabling its proper metallation and disulfide bond formation, thereby ensuring cellular antioxidant capacity [40]. COX17 delivers copper to synthesis of cytochrome oxidase 1/2 (SCO1/2) and cytochrome c oxidase copper chaperone 11 (COX11), which together facilitate the correct maturation and assembly of cytochrome c oxidase subunits COX1 and COX2 [40]. As the terminal complex of the mitochondrial electron transport chain (ETC), cytochrome c oxidase (CCO, also known as complex IV) represents a central hub of energy metabolism and directly determines the efficiency of oxidative phosphorylation and cell survival [41]. Furthermore, ATPase copper transporting alpha (ATP7A) and ATPase copper transporting beta (ATP7B), both P-type copper-transporting ATPases, are responsible for intracellular copper redistribution and efflux to maintain systemic balance [42]. In parallel, glutathione (GSH) and metallothioneins (MTs) act as buffering systems, effectively limiting the accumulation of free copper ions [42]. Through this multilayered regulatory network, copper homeostasis is maintained, ensuring the execution of its physiological functions while preventing toxic effects [42].

When copper homeostasis is disrupted, cells undergo a newly identified form of regulated cell death known as cuproptosis [36]. Unlike apoptosis, necrosis, or ferroptosis, cuproptosis is closely associated with mitochondrial metabolic status and protein lipoylation. In this process, cuprous ions (Cu^+^), under the regulation of ferredoxin 1 (FDX1), bind to lipoylated proteins in the tricarboxylic acid (TCA) cycle, particularly dihydrolipoamide S-acetyltransferase (DLAT), a key enzymatic complex, leading to aberrant protein aggregation and severe proteotoxic stress [43]. Concurrently, copper overload destabilizes iron–sulfur cluster proteins (Fe-S proteins), impairing mitochondrial respiratory chain function, interrupting energy metabolism, and generating large amounts of ROS, thereby reducing mitochondrial membrane potential and ultimately leading to cell death [43]. Notably, cuproptosis does not occur in isolation but intersects with autophagy, ferroptosis, and inflammatory responses [36,44]. For example, copper can regulate autophagy through the reactive oxygen species–mediated AMP-activated protein kinase–mechanistic target of rapamycin (AMPK–mTOR) signaling pathway, or inhibit the activity of GPX4, thereby coupling with ferroptosis, while at the same time enhancing cytokine release by activating inflammation-related signaling pathways [36,44].

To clarify the presentation, we categorize the current evidence supporting the involvement of cuproptosis in spinal cord injury (SCI) into three levels: direct evidence, indirect evidence, and speculative evidence (Table 1). Direct evidence refers to experimental results observed within the context of SCI research that are highly consistent with the typical characteristics of cuproptosis. Indirect evidence primarily reflects disruptions in copper homeostasis following injury, along with biological changes such as mitochondrial dysfunction, enhanced oxidative stress, and exacerbated inflammation. While these changes are not unique to the cuproptosis pathway, they are mechanistically aligned with it. Speculative evidence, on the other hand, primarily comes from transcriptomic or other bioinformatic analyses revealing correlational features, as well as inferences about cuproptosis possibly involving immune regulation or other processes, although there is currently a lack of clear causal validation.

**Table 1.** Direct, indirect, and speculative evidence supporting cuproptosis-related processes in SCI.

Evidence class	Representative evidence/readouts in SCI (as discussed in this review)	Notes/limitations
Direct	SCI-related ischemia reperfusion injury models: copper accumulation accompanied by DLAT oligomerization/aggregation; reduced iron sulfur cluster proteins and lipoylation machinery/readouts (for example, LIAS downregulation); proteotoxic stress responses (for example, HSP70 upregulation); mitochondrial injury readouts (for example, membrane potential decrease and impaired respiration) in the copper-overload context. Copper chelation (for example, ATTM) attenuates these hallmark-consistent changes and is associated with improved histological and functional outcomes.	Direct signatures are mainly from ischemia reperfusion related settings; generalization to traumatic contusion/compression SCI needs caution. Dependence on the canonical FDX1 lipoylation axis remains unproven: definitive genetic evidence (loss of function or gain of function; epistasis or rescue) demonstrating FDX1 or lipoylation dependent vulnerability in copper overload triggered death is still lacking; pharmacologic rescue is not fully specific.
Indirect	Post-SCI copper dyshomeostasis (blood spinal cord barrier disruption, hemorrhage, inflammation) and increased exchangeable copper pool; widespread mitochondrial dysfunction (for example, membrane potential decrease and impaired respiration), oxidative stress (reactive oxygen species surge and glutathione depletion), and inflammatory amplification (for example, the mitochondrial DNA sensing and downstream inflammatory signaling framework discussed in this review). FDX1 expression changes (often downregulation) have also been reported in ischemia reperfusion or hypoxia reoxygenation paradigms.	These are mechanistically compatible but non-specific; mitochondrial injury, oxidative stress, and inflammation are shared across multiple regulated cell death pathways. Notably, FDX1 expression change does not prove FDX1 dependence.
Speculative	Hypothesized roles in immune cell polarization and immune cell cuproptosis; transcriptomic and bioinformatic signatures involving cuproptosis-related genes (for example, SLC31A1, DBT, DLST, LIAS) and immune-associated hubs (for example, CD48, Mpeg1).	Largely association or hypothesis; requires concurrent demonstration in defined cell types of increased copper load, DLAT aggregation, iron sulfur disruption and, critically, FDX1 or lipoylation dependent vulnerability (preferably with genetic perturbation and rescue).

*Notes:* ‘Direct evidence’ refers to combinatorial readouts most consistent with the canonical features of cuproptosis. However, some downstream phenotypes (such as mitochondrial dysfunction, oxidative stress, and inflammation) are not pathway-specific. To date, there is still a lack of genetic causal validation proving that copper overload–induced cell death is strictly dependent on the FDX1–lipoylation axis.

SCI: spinal cord injury; BSCB: blood–spinal cord barrier; I/R: ischemia–reperfusion; H/R: hypoxia–reoxygenation; FDX1: ferredoxin 1; DLAT: dihydrolipoamide S-acetyltransferase; Fe–S: iron–sulfur cluster; LIAS: lipoic acid synthase; ROS: reactive oxygen species; mtDNA: mitochondrial DNA; GSH: glutathione; GPX4: glutathione peroxidase 4; ATTM: ammonium tetrathiomolybdate.

Based on the above classification, current studies indicate that direct evidence mainly originates from ischemia–reperfusion or hypoxia–reoxygenation models of SCI. In contrast, in traumatic contusion or compression models, most observations to date remain within the scope of indirect evidence or speculative associations (Table 1). In the following sections, this framework will be used to systematically review the strength and limitations of existing evidence concerning copper homeostasis alterations, canonical molecular readouts of cuproptosis, and their immunological implications.

In SCI, dysregulation of copper homeostasis and induction of cuproptosis are considered critical molecular mechanisms of secondary damage [36]. Traumatic disruption of the blood–spinal cord barrier (BSCB) permits rapid entry of copper ions into the lesion, while concurrent inflammation and oxidative stress further impair copper transport and buffering systems, resulting in markedly elevated local free copper levels [36,45].

In spinal cord ischemia–reperfusion injury, impaired copper efflux in neurons leads to intracellular copper accumulation, resulting in mitochondrial dysfunction [39,46,47]. This is characterized by a decrease in mitochondrial membrane potential, depletion of glutathione, oligomerization of lipoylated proteins such as DLAT, and downregulation of iron–sulfur cluster proteins such as LIAS, accompanied by elevated expression of the stress marker HSP70 [39,46,47]. Together, these molecular and functional alterations drive neuronal death and tissue damage. These research findings show a high level of consistency with the key characteristics of cuproptosis, making them one of the relatively clear direct pieces of evidence in current spinal cord injury research (Table 1).

**Figure 1.**
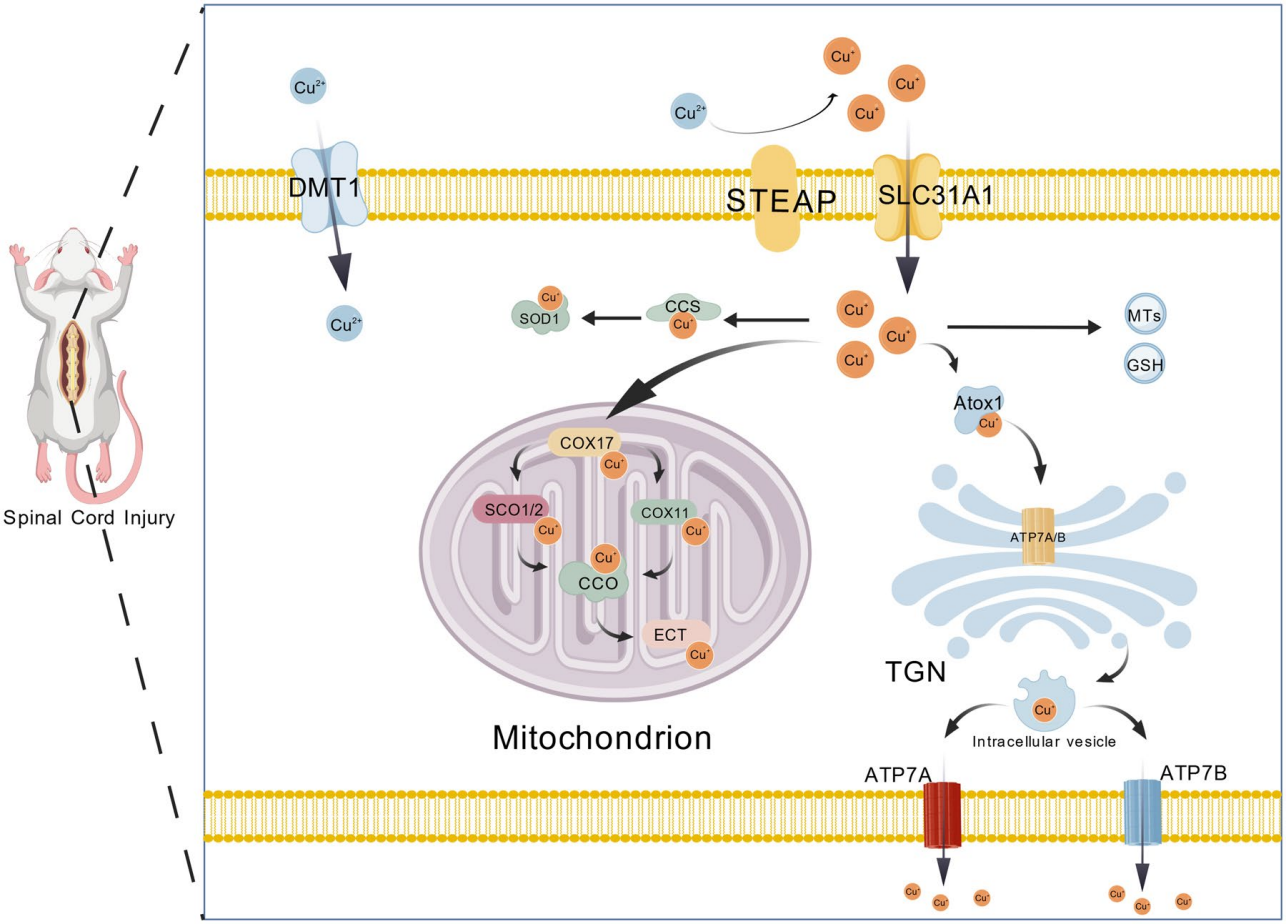
Overview of cellular copper metabolism and homeostasis. Dietary cupric ions (Cu^2+^) are reduced to cuprous ions (Cu^+^) by STEAP metalloreductases and imported into intestinal epithelial cells *via* CTR1 or divalent metal transporter 1 (DMT1). Within the cytosol, Cu^+^ is specifically delivered by metallochaperones: ATOX1 transfers copper to the P-type ATPases ATP7A and ATP7B for intracellular redistribution and efflux; CCS supplies Cu^+^ to Cu/Zn-SOD1 to support antioxidant defense; and COX17 delivers copper to SCO1/2 and COX11 to enable the proper assembly of cytochrome c oxidase (CCO, complex IV). As the terminal complex of the mitochondrial electron transport chain, CCO serves as a central hub of energy metabolism, directly determining oxidative phosphorylation efficiency and cell survival. Excess Cu^+^ is transiently buffered by glutathione (GSH) and metallothioneins (MTs) to prevent cytotoxicity. Created with BioGDP.com [122]. STEAP: six-transmembrane epithelial antigen of the prostate; CTR1: Cu transporter 1; DMT1: divalent metal transporter 1; Cu: copper; ATOX1: antioxidant protein 1; ATP7A: Cu-transporting ATPase 1; ATP7B: ATPase Cu-transporting beta; CCS: copper chaperone for superoxide dismutase; SOD1: superoxide dismutase 1; COX17: cytochrome c oxidase copper chaperone 17; SCO1/2: synthesis of cytochrome c oxidase 1/2; COX11: cytochrome c oxidase assembly factor 11; CCO: cytochrome c oxidase (complex IV); ETC: electron transport chain; GSH: glutathione; MTs: metallothioneins; LOX: lysyl oxidase; CP: ceruloplasmin; TGN: trans-Golgi network.

In addition, copper-driven microglial activation and pro-inflammatory cytokine release further amplify local inflammation, creating a vicious cycle that culminates in axonal degeneration and neurological dysfunction [36,48]. Collectively, disruption of copper homeostasis and activation of cuproptosis act synergistically and mutually reinforcingly in the pathogenesis of SCI, constituting a crucial component of secondary injury and providing a novel theoretical framework for elucidating SCI mechanisms and identifying potential therapeutic targets (Figure 1).

### 4.1. Copper homeostasis imbalance and the formation of a pro-cuproptosis microenvironment

After spinal cord injury, the structural integrity of the blood–spinal cord barrier is compromised, and local microcirculatory perfusion becomes impaired. These factors together affect the entry, distribution, and clearance of metal ions in the injured area [49,50]. Due to local hemorrhage, edema, and disruptions in ion exchange processes, copper tends to accumulate more easily in the core and surrounding tissues of the injury [49]. Additionally, secondary inflammatory responses and oxidative stress continue to deplete intracellular reducing agents such as glutathione, while interfering with the homeostasis of copper-binding proteins like metallothioneins, leading to an increased intracellular copper pool capable of participating in reactions [49,50].

Copper exhibits significant redox activity. When free copper levels rise, it not only promotes the generation of reactive oxygen species but may also disrupt the structural stability of proteins involved in mitochondrial energy metabolism [51]. These changes further weaken the protein homeostasis regulatory mechanisms, making neurons, glial cells, and infiltrating immune cells more susceptible to copper-induced toxic stress, thereby providing a backdrop for the initiation of copper death-related molecular reactions [51–53].

From a cellular regulation perspective, the copper efflux mechanism plays a key role in limiting copper accumulation within cells [39]. Studies have shown that during spinal cord ischemia-reperfusion injury, if copper efflux is restricted, the copper load in tissues significantly increases. This change is often accompanied by abnormal deposition of acylation substrates, impaired function of iron-sulfur cluster proteins, and enhanced mitochondrial stress. It is noteworthy that interventions aimed at reducing copper load can alleviate tissue damage and improve neurological dysfunction [39]. This phenomenon suggests that disturbances in copper homeostasis may participate in the activation of cellular damage mechanisms in the early stages of the pathological process and further drive injury progression by triggering copper death-related events [39].

Existing animal experimental evidence has revealed significant changes in copper load at the tissue level after spinal cord injury. For example, in a rat spinal cord injury model, the local copper content was found to be significantly elevated in the injured spinal cord tissue homogenates, accompanied by increased oxidative stress levels [54]. These results indicate that copper accumulation may play a pathological role following injury. However, it is important to note that differences in experimental model selection, sampling regions, and time points across studies can affect the comparability and interpretability of results.

Regarding quantitative studies of post-injury copper load changes, there are differences in model types, sampling levels, and time windows across various studies. In existing research, total copper content in tissue or sorted cell samples can be measured using inductively coupled plasma mass spectrometry (ICP-MS) [53]. The spatial distribution of metals in the injury site and surrounding regions can be analyzed using laser ablation coupled with mass spectrometry imaging [55]. Dynamic changes in the exchangeable copper pool can also be visualized using copper-responsive fluorescent probes combined with imaging strategies [56]. Although methodological differences limit direct comparisons between studies, the existing evidence generally supports a consistent pathological trend between increased copper load in the injury region and enhanced copper toxicity signaling, providing an important foundation for further exploring the interaction between cuproptosis and the immune microenvironment [57].

Currently, although traumatic contusion or compression models are widely used in SCI mechanism research, specific changes in copper content in the injury area and its surrounding tissues, particularly the dynamic changes within different cell types, still lack systematic data support. To better understand the formation process of the pro-cuproptosis-related pathological environment, future research should integrate tissue-level elemental quantification with the distribution characteristics of copper within tissues, based on clear experimental designs, to more comprehensively reveal the role of copper metabolic abnormalities in the pathological process.

### 4.2. Cuproptosis-Associated mitochondrial damage and enhanced inflammatory response

Cuproptosis is a form of regulated cell death closely related to the mitochondrial metabolic state. In the classical mechanism model, its occurrence is thought to be closely associated with key regulatory factors such as FDX1 and various acylation-related genes (e.g. LIAS, LIPT1, DLD, and DLAT). In spinal cord injury models, changes in FDX1 expression have been observed, such as a downregulation following spinal cord ischemia-reperfusion injury or hypoxia-reoxygenation treatment, suggesting its potential involvement in the cuproptosis process. However, whether FDX1 determines the cell death induced by copper overload (i.e. whether there is an explicit FDX1/acylation axis dependency) still lacks direct genetic causal evidence (such as knockout/overexpression with rescue or upstream-downstream epistasis verification), meaning this part remains more of an indirect clue [39].

Under conditions of increased intracellular copper load, copper ions bind to acylation substrates in the tricarboxylic acid cycle, inducing abnormal protein aggregation while disrupting the stability of iron-sulfur cluster proteins. This leads to protein toxicity stress, mitochondrial dysfunction, and ultimately, cell death [58]. In spinal cord ischemia-reperfusion injury models, impaired copper efflux and intracellular copper accumulation are often accompanied by changes such as DLAT oligomerization, reduced iron-sulfur cluster proteins, and enhanced mitochondrial stress, all of which are highly consistent with the typical molecular characteristics of cuproptosis [58]. Studies have shown that reducing copper load with copper chelators can effectively alleviate mitochondrial stress, improve tissue structure, and enhance neurological function recovery, suggesting that copper homeostasis disruption plays a crucial role in secondary tissue injury by activating the cuproptosis mechanism [39].

**Figure 2.**
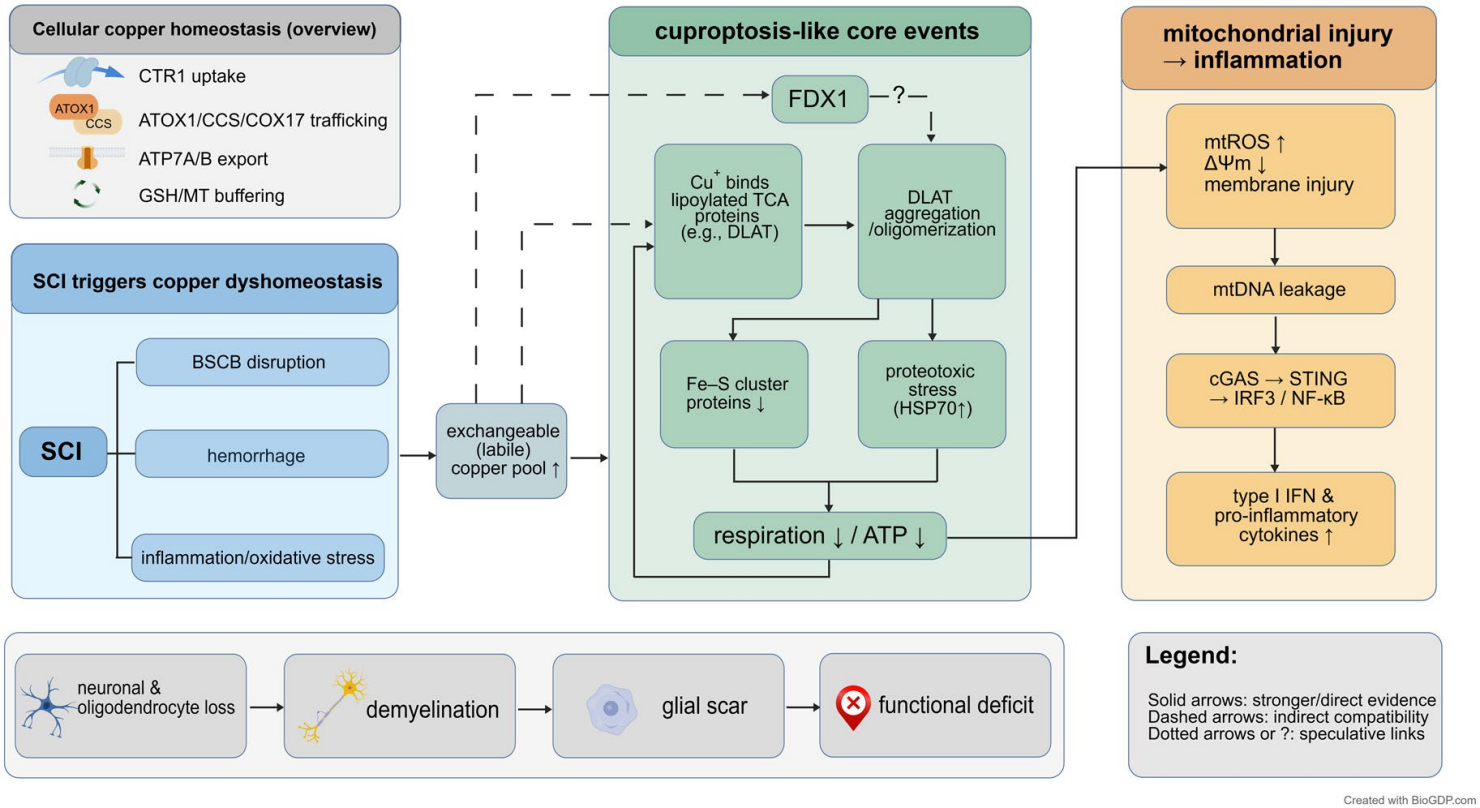
Copper dyshomeostasis promotes mitochondrial stress and inflammation after SCI. Spinal cord injury can lead to increased levels of exchangeable copper in the injured region through disruption of the blood–spinal cord barrier (BSCB), hemorrhage, inflammation, and oxidative stress. The elevated copper load interacts with acylated proteins involved in mitochondrial metabolism, inducing protein aggregation, destabilization of iron–sulfur (Fe–S) cluster proteins, and impaired respiration and energy production. These changes contribute to mitochondrial damage and the release of mitochondrial DNA (mtDNA) into the cytoplasm. The leaked mtDNA can activate the cGAS–STING signaling pathway and enhance pro-inflammatory transcription, thereby amplifying the secondary inflammatory response. Solid lines indicate well-supported relationships, dashed lines represent indirect or consistent associations, and dotted lines or question marks denote speculative links. Created with BioGDP.com [122]. SCI: spinal cord injury; BSCB: blood–spinal cord barrier; DLAT: dihydrolipoamide S-acetyltransferase; Fe–S: iron–sulfur cluster; mtDNA: mitochondrial DNA; cGAS: cyclic GMP–AMP synthase; STING: stimulator of interferon genes; NF-κB: nuclear factor kappa B.

Mitochondrial damage not only affects cellular energy metabolism but also can trigger a series of immunogenic signals, driving the sustained activation of local inflammatory responses [59]. After neurons or glial cells undergo mitochondrial stress associated with copper overload, they typically experience an accumulation of reactive oxygen species (mtROS), a decline in mitochondrial membrane potential, and membrane structure disruption, significantly increasing the risk of mitochondrial DNA (mtDNA) leakage into the cytoplasm [60]. Cytoplasmic mtDNA can be recognized by cGAS, activating the STING pathway and triggering an inflammatory transcriptional program mediated by IRF3 and NF-κB. This leads to enhanced expression and secretion of pro-inflammatory cytokines and promotes inflammasome activation, further exacerbating local tissue damage. The activation of this nucleic acid sensing pathway forms a crucial bridge between mitochondrial dysfunction and immune amplification [60]. The progression from SCI-induced disruption of copper homeostasis to mitochondrial stress resembling cuproptosis, followed by the release of mtDNA and activation of the cGAS–STING inflammatory pathway, is summarized in Figure 2.

Multiple studies have confirmed the feasibility of targeting this process therapeutically. For instance, the mitochondria-targeted antioxidant Mito-TEMPO has been shown to effectively scavenge mitochondrial reactive oxygen species (mtROS), reduce mtDNA leakage, and attenuate downstream inflammatory phenotypes [61]. Inhibitors of the STING pathway, such as C-176 or siRNA, can block the activation of this signaling cascade and thereby alleviate inflammation [62]. Moreover, preserving mitochondrial structural integrity is also critical for preventing aberrant activation of nucleic acid-sensing pathways. The mitochondrial fusion protein Mfn2 plays a key role in maintaining mitochondrial architecture and limiting mtDNA release. Studies have found that reduced Mfn2 expression increases the susceptibility of cGAS–STING pathway activation and promotes microglial hyperactivation. In contrast, the Mfn2 agonist MASM7 can promote mitochondrial fusion and suppress inflammatory cascades [63].

With regard to copper homeostasis, copper chelators such as ammonium tetrathiomolybdate (ATTM) have been shown to partially restore intracellular glutathione levels, reduce mtROS production, and maintain mitochondrial function. These effects collectively inhibit NF-κB activation and suppress the expression of multiple pro-inflammatory cytokines, thereby mitigating inflammation [64]. Collectively, these findings highlight the central role of cuproptosis-associated mitochondrial damage in driving inflammatory amplification and suggest potential therapeutic targets for intervening in the secondary pathological processes following SCI.

### 4.3. The impact of copper-induced metabolic stress on immune cell polarization

Following spinal cord injury (SCI), microglia and infiltrating peripheral macrophages serve as key effector cells in shaping the immune microenvironment. Their dynamic phenotypic transitions directly influence the extent of inflammation and the outcomes of tissue repair [65]. Previous studies have shown that microglia tend to adopt a pro-inflammatory phenotype upon exposure to inflammatory stimuli, releasing various cytokines that exacerbate neuronal injury and axonal degeneration. However, under the regulation of specific cytokines, they can also shift toward a more reparative phenotype, contributing to phagocytosis and tissue reconstruction [65]. Under conditions of copper dyshomeostasis, copper-induced metabolic stress may influence microglial polarization through both intercellular signaling and intrinsic regulatory mechanisms [65].

Signals derived from neighboring damaged cells can continuously drive microglia toward a pro-inflammatory state [66]. During mitochondrial stress and cellular injury induced by copper accumulation, neurons and glial cells may release stress-associated products such as nucleic acid fragments, which in turn activate intracellular nucleic acid-sensing and inflammatory transcription pathways. This enhances the expression of pro-inflammatory cytokines and prolongs the duration of the inflammatory state [3,60,67]. Previous studies have shown that under conditions of mitochondrial DNA leakage, activation of the cGAS–STING signaling pathway promotes microglial activation and significantly contributes to the severity of post-injury inflammation [63].

At the gene regulation level, recent studies have found that Mpeg1 is upregulated in microglia following SCI and is associated with both activation status and signals related to tissue repair. *In vitro* experiments have shown that silencing Mpeg1 significantly increases the secretion of TNF-α and IL-1β, along with elevated levels of pro-inflammatory markers [68]. Considering the role of Mpeg1 in maintaining phagocytic function and lysosomal homeostasis, its upregulation may help clear cellular stress products, alleviate sustained mitochondrial stress signaling, suppress pro-inflammatory polarization, and promote a more repair-supportive immune state [69]. Nevertheless, the specific relationships among copper load, mitochondrial function, and cuproptosis-related markers still require single-cell level validation to clarify their roles in copper-induced metabolic stress [68].

In summary, constructing a mechanistic framework that integrates sustained external signaling with intracellular copper-induced metabolic stress provides a basis for understanding how cuproptosis influences immune polarization. This approach may help delineate research directions and facilitate the translation of this complex process into feasible and testable experimental models.

### 4.4. Potential role of cuproptosis in immune cells and its Impact on immune lineage regulation

Following spinal cord injury (SCI), changes in the immune microenvironment are not only driven by intercellular signaling that promotes immune activation but may also be closely related to changes in the fate of immune cells themselves. On one hand, neurons and glial cells undergoing mitochondrial stress associated with cuproptosis can release mitochondrial-derived danger signals, such as mtDNA and TFAM [70]. These signals can activate microglia and further amplify neuroinflammatory responses. This process not only exacerbates local inflammation but also promotes the recruitment and infiltration of peripheral immune cells, forming a self-perpetuating inflammatory cycle that may contribute to neurodegeneration and ongoing tissue damage [71].

On the other hand, immune cells themselves, under conditions of high copper load and metabolic stress, may experience mitochondrial dysfunction and initiate the cuproptosis pathway, thereby directly impacting cell survival and altering the composition of immune subpopulations [72].

In the context of adaptive immunity, the proliferation and effector functions of activated T cells rely on the coordination between glycolysis and oxidative metabolism, whereas regulatory T cells (Tregs) primarily depend on oxidative phosphorylation and fatty acid oxidation to maintain their immunosuppressive functions [73]. Under conditions of copper overload, copper can bind lipoylated DLAT, inhibit pyruvate dehydrogenase activity, disrupt mitochondrial metabolism and energy homeostasis, and thereby potentially affect T-cell function and fate decisions [74^,^^75^]. In SCI samples, increased infiltration of Treg cells has been observed, accompanied by dynamic changes in the composition of myeloid cells. This suggests that the remodeling of immune cell populations may be co-regulated by metabolic stress and the inflammatory microenvironment [76,77].

Specifically, in SCI tissue, Treg cell infiltration is significantly increased, while multiple effector T cell subsets exhibit reduced numbers or altered functionality [78,79]. Concurrently, the myeloid compartment, including macrophages and microglia, also undergoes notable changes [1]. These findings indicate that the reconstruction of the immune landscape may be shaped by both local inflammatory cues and copper-related metabolic pressure [80]. The increase in Treg cells likely reflects a compensatory immunoregulatory response, while the decrease in effector T cells could result from suppression of inflammation or metabolic disruption [79]. Additionally, altered myeloid cell function further influences tissue repair and regeneration after injury [1]. However, changes in immune cell populations could also result from impaired migration, reduced proliferation, or other forms of cell death, and do not alone confirm that cuproptosis has occurred.

To determine whether immune cells undergo cuproptosis, it is necessary to integrate measurements of intracellular copper accumulation, the activation of key molecular events, and reproducible changes in cell fate or function. Representative functional outcomes may include reductions in specific immune subsets, decreased mitochondrial respiration, imbalanced inflammatory or regulatory functions, and impaired phagocytosis or antigen presentation. Future studies could isolate microglia, infiltrating macrophages, and different T cell subsets from animal models and quantify intracellular copper levels, aggregation of acylated proteins, stability of iron–sulfur cluster proteins, mitochondrial membrane potential, and respiratory capacity. Furthermore, genetic or pharmacological interventions targeting FDX1 or acylation-related pathways, along with copper chelator treatments, can help assess whether the observed cell death and dysfunction are dependent on the cuproptosis pathway.

**Figure 3.**
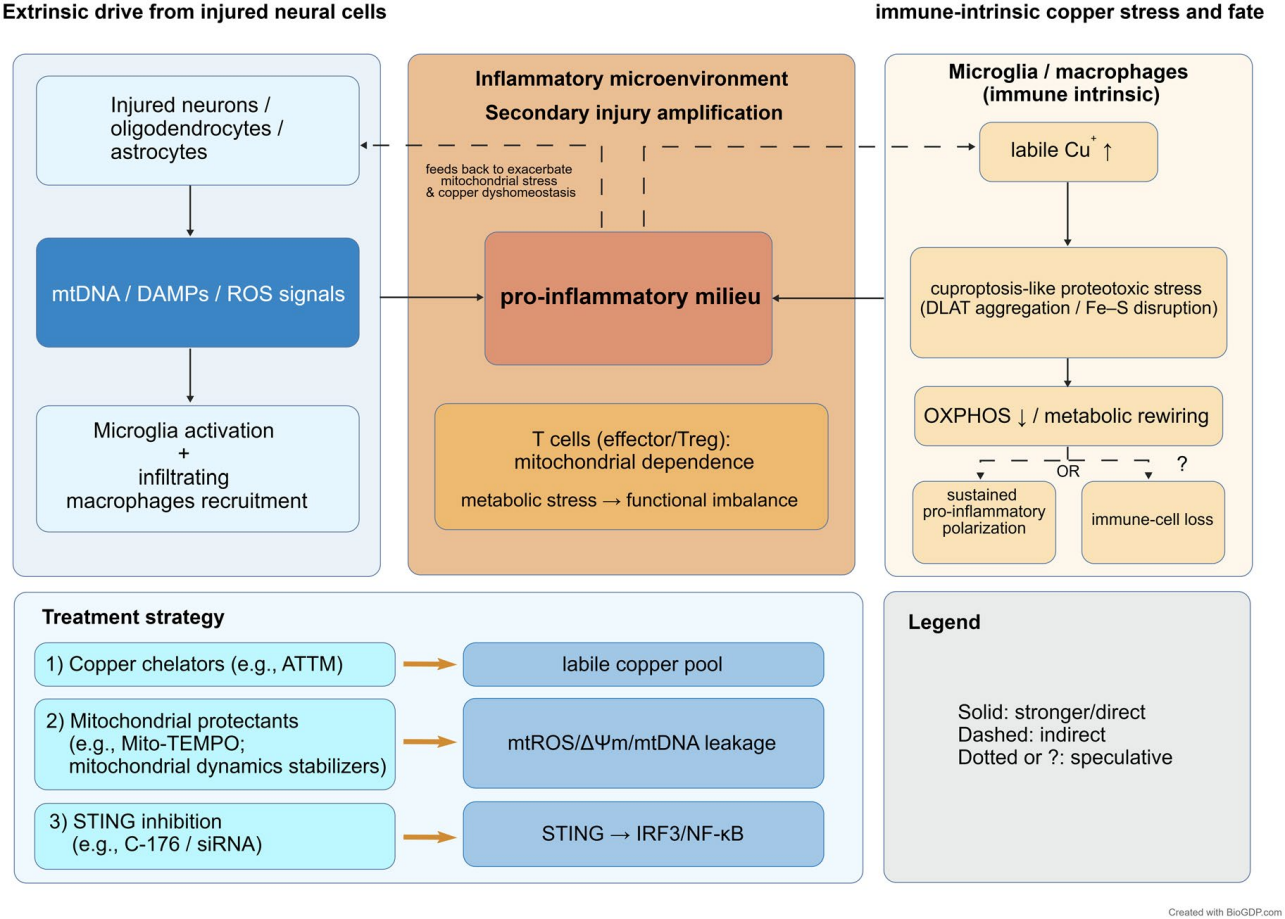
Cuproptosis-related immune dysregulation in SCI. Danger signals released by injured cells can promote microglial activation and the recruitment of peripheral macrophages, thereby exacerbating the inflammatory microenvironment and contributing to secondary injury. Elevated intracellular copper levels within immune cells may impair mitochondrial metabolism and induce protein toxicity stress, which can sustain pro-inflammatory phenotypes or lead to immune cell loss. Additionally, this may disrupt the functional balance of T cells. Intervention strategies such as copper chelation, mitochondrial protection, and STING inhibition are indicated at their respective sites of action. Different line styles are used in the figure to distinguish between well-supported evidence, indirect associations, and speculative links. Created with BioGDP.com [122]. DAMPs: damage-associated molecular patterns; mtDNA: mitochondrial DNA; OXPHOS: oxidative phosphorylation; Treg: regulatory T cell; ATTM: ammonium tetrathiomolybdate; STING: stimulator of interferon genes.

If increased copper burden, activation of cuproptosis-related markers, and corresponding changes in cell fate or function can be simultaneously observed in immune cells, and these alterations are correlated with post-injury immune lineage remodeling, this would provide strong evidence for the involvement of cuproptosis in reshaping the immune microenvironment. Such findings would also support the development of therapeutic strategies integrating copper homeostasis regulation with immune modulation. Based on these considerations, Figure 3 summarizes the pro-inflammatory signals released by damaged cells, copper-related metabolic stress within immune cells, and their combined effects on the inflammatory microenvironment, with representative therapeutic targets highlighted.

## 5. Discussion

### 5.1. Clinical observations of copper homeostasis alterations after spinal cord injury

Following spinal cord injury (SCI), the body enters a stress state primarily characterized by inflammation and tissue repair [81]. SCI can trigger an acute-phase response in the liver, leading to alterations in the synthesis and secretion of acute-phase proteins and certain metal-related proteins, such as ceruloplasmin and metallothioneins [82–85]. Copper in peripheral blood primarily exists in a protein-bound form, so changes in total serum copper and copper-related proteins often correlate with the systemic response to injury [86].

Current clinical studies suggest that in patients with acute traumatic SCI, the levels of total copper and copper-related proteins, particularly ceruloplasmin, change over the course of the injury and may be associated with subsequent neurological recovery [49]. In one prospective cohort study, serum total copper and ceruloplasmin levels were sampled at multiple time points within 24 h of hospital admission. The results showed that patients who later exhibited improvements in AIS (American Spinal Injury Association Impairment Scale) grade had relatively higher ceruloplasmin levels upon admission, along with a more pronounced decline in both total copper and ceruloplasmin levels during the initial 24 h [49]. These findings suggest that copper metabolism-related phenotypes may reflect the intensity and dynamic changes of the systemic response following SCI and could potentially serve as indicators for prognosis [49].

Beyond acute-phase evidence, early epidemiological studies have also observed differences in plasma copper levels between SCI patients and healthy individuals [87]. This suggests that disturbances in copper homeostasis may not be confined to the acute traumatic phase and could be influenced by the type of injury and clinical context [49]. It is important to note that peripheral copper and ceruloplasmin levels can be influenced by disease stage, inflammation or infection status, liver function, and nutritional conditions [88]. Therefore, when comparing studies, a multifactorial assessment should be conducted to avoid misinterpreting the clinical significance based on fluctuations in a single parameter [88].

Compared to blood-based indicators, quantitative studies of copper and its related proteins in cerebrospinal fluid (CSF) remain limited. Current CSF biomarker research primarily focuses on markers such as neurofilament light chain (NFL) and glial fibrillary acidic protein (GFAP), which are used to assess injury severity and predict outcomes [89]. In contrast, the patterns of change, influencing factors, and clinical significance of copper and copper-related proteins in CSF have not been fully elucidated. Future studies could build on prospective cohorts with standardized sampling and analytical conditions to systematically accumulate data and compare CSF and peripheral blood indicators to enhance the interpretability and clinical applicability of the results [90].

### 5.2. Preclinical evidence and translational challenges of copper homeostasis targeted intervention

Preclinical studies on the regulation of copper homeostasis after spinal cord injury (SCI) have primarily developed two intervention strategies. One approach aims to reduce abnormal bioavailable copper levels after injury, thereby alleviating copper-related mitochondrial metabolic stress and cellular toxicity, leading to neuroprotective effects. The other approach involves utilizing copper-containing delivery systems or functional materials to improve the local microenvironment at the injury site, alleviating secondary injury through anti-inflammatory and antioxidant effects and promoting repair.

In research focused on limiting bioavailable copper, spinal cord ischemia-reperfusion injury models have provided experimental evidence supporting the involvement of copper-dependent cell death in the injury process. Studies in animal and cell models have shown that the copper efflux transporter ATP7B is downregulated, accompanied by increased intracellular copper load and abnormalities in acylated proteins [39]. When treated with the copper chelator ammonium tetrathiomolybdate (ATTM), neuronal damage was reduced, cell survival improved, and hind limb motor function showed improvement. These findings suggest that limiting bioavailable copper may coincide with neuroprotection, providing direct evidence for copper homeostasis as an intervention target [39].

From a translational perspective, copper chelators have relatively mature clinical applications in diseases such as Wilson’s disease, which involves copper overload, thus offering valuable insights into dosage regulation, efficacy monitoring, and adverse effect management [91]. Moreover, tetrathiomolybdate-class copper chelation has recently been linked to modulation of cuproptosis-associated signaling in other disease contexts (e.g. PI3K/Akt–related mechanisms), reinforcing both the therapeutic relevance and the need to carefully evaluate systemic pathway effects when repurposing chelation strategies for SCI [92]. Since copper plays a fundamental role in energy metabolism and antioxidant defense, a more optimal strategy may be to limit the pro-damaging effects of excess reactive copper, rather than continuously lowering overall copper levels in the body [91]. Considering this, future pharmacological research should more precisely define target ranges and clarify the impact of interventions on copper species and subcellular distribution, in order to minimize the risk of metabolic burden due to copper deficiency.

In experimental models of traumatic spinal cord injury, researchers more commonly use local delivery methods combined with biomaterial strategies to enhance therapeutic effects and improve drug targeting at the injury site. For example, chitosan-derived injectable hydrogels have been developed, in which copper ions are coordinated to form crosslinked structures. These hydrogels also incorporate triptolide to enhance the response to the inflammatory environment [93]. Animal experiments have shown that this hydrogel reduces inflammation, improves the function of neuron-associated cells, enhances motor scores, and promotes axon regeneration [93]. Copper-catalyzed materials have also been used to reduce oxidative stress and inflammation, thus improving the post-injury microenvironment. For instance, platinum-copper nanzymes wrapped in bovine serum albumin promote hydrogen peroxide clearance, reduce reactive oxygen species (ROS), and alleviate mitochondrial dysfunction, with observed improvements in motor function [94]. Researchers have suggested that this effect may be related to the regulation of the mitochondrial damage process mediated by BNIP3 [94]. Another study developed copper-based materials loaded with ruthenium clusters, which were found to clear ROS, reduce inflammation, and promote repair in inflammation-related spinal cord injury models [95]. More recently, a multifunctional hybrid neural restorative conduit was developed to achieve spatiotemporal release of multiple bioactive factors (including catalase for oxidative stress control and neurotrophic cues such as NT3 and GDNF), thereby remodeling the hostile post-SCI microenvironment and improving neuronal survival, axonal relay, motor recovery, and urinary control *in vivo* [96].

In addition to copper load regulation and material strategies, some studies have also attempted to achieve neuroprotection by modulating copper-dependent cell death pathways. For example, a recent study reported that neurotrophin-3 (NT3)-loaded chitosan could modulate cuproptosis-related genes in SCI and promote functional recovery, supporting the feasibility of targeting cuproptosis-linked molecular programs *via* local biomaterial-based delivery [6]. This phenomenon suggests that some interventions may reduce cell susceptibility to copper stress through molecular regulatory processes, without necessarily relying on changes in copper concentration itself [6].

Additionally, nutritional supplementation strategies have been explored in experimental research. One animal study evaluated the effects of vitamin E combined with zinc, selenium, and copper supplementation, showing improvements in immune-related markers and accompanying motor function enhancement [97]. However, due to the inclusion of multiple components in the intervention, the independent contribution of copper cannot be separated from this design, making it more suitable for suggesting the potential value of integrated nutritional support, rather than directly demonstrating the specific therapeutic effects of copper-targeted intervention [97].

Overall, current preclinical studies support the involvement of copper homeostasis in secondary injury and repair processes after SCI, but a systematic and reproducible causal evidence chain is still lacking. Future work should, in addition to functional and pathological evaluations, simultaneously measure copper load at the injury site and in body fluids, quantify changes in copper transporters and chaperone proteins, and analyze copper-dependent cell death signaling pathways. By linking copper load, key molecular changes, and functional outcomes within the same research framework, this will help clarify the role of copper homeostasis alterations in the repair process and provide interpretable mechanistic evidence for future translational research.

### 5.3. Feasibility of cuproptosis as a diagnostic and prognostic biomarker

Cuproptosis has the potential to serve as a biomarker because it is often accompanied by disruptions in copper homeostasis, impaired mitochondrial metabolism, and changes in the stability of acylated proteins [98]. Once copper accumulates within cells, it interacts with acylated proteins involved in the tricarboxylic acid cycle, leading to protein aggregation. It can also induce dysfunction of iron-sulfur cluster proteins and a protein toxicity stress response, ultimately driving cell death [98]. Since these pathological processes leave various signals in body fluids, peripheral immune cell expression profiles, and local metabolic stress responses at the injury site, a comprehensive evaluation of multiple readouts is more likely to reflect the full pathological evolution following injury [99].

Spinal cord injury (SCI) biomarker research often needs to balance early risk prediction with outcome forecasting. Based on the clinical observations mentioned earlier, fluid markers such as total serum copper and ceruloplasmin are accessible for detection and could serve as candidate indicators for early risk assessment [86]. However, their fluctuations are easily influenced by systemic inflammatory responses, acute-phase reactions in the liver, and factors such as infection and nutritional status. Therefore, these markers are better suited as risk indicators and should be interpreted in conjunction with molecular-level readouts [86]. By incorporating molecular signals that reflect mitochondrial metabolic stress and changes in immune status alongside copper-related indicators in body fluids, we can provide supplementary clues to better understand the secondary damage processes following injury [86].

Recent transcriptomic studies have provided specific insights into this direction. One study, based on publicly available peripheral sample data, screened candidate genes and proposed a model composed of SLC31A1, DBT, DLST, and LIAS to distinguish injury status, which was later validated by animal experiments showing consistent trends in gene expression changes [100]. Another study used co-expression analysis combined with immune infiltration correlation analysis to identify hub genes. It reported that candidate molecules such as CD48 and Mpeg1 are closely associated with immune processes and observed enhanced expression of Mpeg1 in microglia following injury in single-cell data [68]. These results suggest that some copper-dependent cell death-related candidate genes may appear alongside immune cell activation and inflammatory response evolution, making them promising candidates for reflecting pathological states and providing stratification clues [68].

In addition to peripheral transcriptomic characteristics, preclinical studies also suggest that relevant molecular readouts may change with disease progression and intervention response, with their trends often aligning with tissue damage severity and motor outcomes. Therefore, these markers can be considered candidates for pharmacodynamic indicators and prognostic assessment [39]. However, current evidence is primarily based on retrospective analyses and bioinformatic inferences, and several key issues need to be addressed before clinical application [68]. Study designs should standardize sampling windows and include longitudinal follow-up to reduce inconsistencies caused by differences in disease stages [101]. Peripheral blood testing should sufficiently control for confounding factors such as infection, liver dysfunction, and nutritional interventions, while systematically recording and adjusting for key covariates to improve the reproducibility and comparability of results. Cerebrospinal fluid (CSF) is closer to the central injury process, but existing quantitative evidence on copper and its related proteins remains limited. Therefore, data should be systematically accumulated under standardized collection and testing conditions, and comparative analysis between CSF and peripheral markers should be conducted to clarify its added value. Since single markers often lack both specificity and robustness, a more preferable strategy is to integrate copper homeostasis-related markers in body fluids with copper-dependent cell death-related molecular features into a unified analysis framework, alongside existing neuroinjury biomarkers and clinical scales. This approach would help assess their independent contribution to outcome prediction and enhance the interpretability of risk stratification.

In summary, Cuproptosis-dependent markers are better suited as candidates for stratification and dynamic monitoring. Future studies should rely on prospective cohorts with longitudinal observation to clarify their dynamic patterns and the impact of confounding factors, and test their independent associations with functional outcomes, thereby laying the foundation for translational application.

### 5.4. The relationship and boundaries between cuproptosis, ferroptosis, and pyroptosis in the immune microenvironment of spinal cord injury

After spinal cord injury (SCI enters the secondary injury phase, local tissues are often accompanied by disruptions in metal ion homeostasis, mitochondrial dysfunction, and sustained enhancement of inflammatory signaling [102]. These pathological changes may provide favorable conditions for multiple forms of programmed cell death to occur simultaneously within the same lesion [102]. As such, cuproptosis, ferroptosis, and pyroptosis mechanisms may not only overlap temporally but could also appear spatially in different cell types [103]. To avoid misinterpreting the co-activation of multiple pathways as the same form of cell death, it is important to distinguish the induction backgrounds, key execution steps, and immune consequences of each mechanism [103].

The core feature of cuproptosis is its high dependence on mitochondrial respiratory function and tricarboxylic acid (TCA) cycle activity [103]. When intracellular copper levels rise, copper ions preferentially bind to acylated TCA cycle-related enzymes, inducing abnormal protein aggregation [104]. This process not only disrupts the homeostasis of iron-sulfur cluster proteins but also triggers protein toxicity-related cellular stress responses, leading to sustained metabolic pressure accumulation. As protein homeostasis deteriorates, mitochondrial function gradually fails, ultimately causing cell death [104]. Therefore, the key pathogenic steps of cuproptosis primarily lie in the disruption of metabolic protein homeostasis and the linked mitochondrial damage process [104].

Ferroptosis, on the other hand, is characterized by uncontrolled membrane lipid peroxidation [105]. After tissue injury, associated hemorrhage and increased intracellular iron load promote reactive oxygen species (ROS) production, expanding the supply of polyunsaturated fatty acid phospholipids as peroxidation substrates [105]. When the antioxidant capacity of glutathione decreases, or when glutathione peroxidase 4 (GPX4) function is limited, the cell’s ability to clear lipid peroxides significantly diminishes, leading to irreversible membrane damage. In this process, the continuous accumulation of iron-dependent lipid peroxides constitutes the core of ferroptosis, while membrane damage becomes the direct driver of cell death [105].

Pyroptosis typically begins with inflammasome activation, which triggers the cleavage of gasdermin family members, releasing their pore-forming domains [106]. These domains insert into the cell membrane, forming permeable pores that compromise membrane integrity, resulting in imbalanced ion flux and cell swelling [106]. Pore formation also promotes the release of pro-inflammatory cytokines, such as interleukin-1β and interleukin-18. Thus, the key execution steps of pyroptosis include inflammasome-mediated pore formation and active release of inflammatory factors, which are particularly prominent during the acute injury phase, helping to rapidly amplify the inflammatory response [106].

Both cuproptosis and ferroptosis share certain similarities in the immune microenvironment following SCI, the most notable of which is their susceptibility to amplification by redox imbalance [36]. During cuproptosis, abnormal protein aggregation and disruption of iron-sulfur cluster homeostasis significantly increase mitochondrial oxidative stress, leading to elevated ROS levels [107]. The accumulation of ROS not only exacerbates mitochondrial damage but also promotes the formation of lipid peroxidation substrates, thereby increasing the oxidative load required for ferroptosis induction [107]. Although both cuproptosis and ferroptosis are regulated by oxidative stress, they differ fundamentally in their death mechanisms [108]. Cuproptosis relies primarily on the sustained disruption of mitochondrial protein homeostasis, while ferroptosis is closely linked to the reduced ability of cells to clear lipid peroxides [108]. Therefore, oxidative stress should be understood as a shared upstream amplifier, rather than a distinguishing factor for cell death type [107].

Copper accumulation may also enhance cellular susceptibility to ferroptosis by interfering with GPX4 homeostasis [44]. Recent studies suggest that copper-induced cellular stress can activate selective autophagy pathways, leading to the degradation of GPX4 and impairing the cell’s ability to clear lipid peroxides [44]. If copper homeostasis imbalance, elevated lipid peroxidation levels, and reduced GPX4 expression are observed simultaneously, these changes are more likely to reflect the weakening of antioxidant defenses caused by copper-related metabolic stress, thus promoting the onset of ferroptosis [44]. To accurately identify this complex mechanism, key evidence should focus on the aggregation of acylated proteins and the accumulation of lipid peroxides, in order to determine whether cuproptosis and ferroptosis jointly occur in the lesion, rather than interpreting them as one and the same.

Ferroptosis plays an amplifying role in regulating the immune microenvironment following SCI, particularly evident in the pathological changes of the blood-spinal cord barrier (BSCB)[109]. Studies in animal contusion models have shown that inhibiting ferroptosis can effectively reduce endothelial cell damage, maintain the structural and functional integrity of the BSCB, and significantly decrease the recruitment of inflammatory cells and overactive glial cell responses [110]. This suggests that ferroptosis in endothelial cells may compromise barrier function, allowing peripheral immune cells to enter the injury site [110]. Subsequently, these infiltrating immune cells release inflammatory mediators, increasing oxidative stress, which further induces ferroptosis, creating a sustained positive feedback loop [109]. Further animal experimental evidence also suggests that the source of iron may be closely linked to the phagocytosis and breakdown of red blood cells following injury [111]. Additionally, tissue damage caused by ferroptosis can extend into the subacute and chronic stages, indicating that intervening in this process over longer treatment durations may improve functional recovery outcomes [110].

While cuproptosis and pyroptosis have overlapping initiation mechanisms, key differences in their execution phases remain evident. Pyroptosis is heavily dependent on inflammasome activation and subsequent gasdermin-mediated pore formation, whereas cuproptosis does not rely on this process [112]. However, copper-induced mitochondrial stress can significantly enhance the release of danger-associated molecular patterns (DAMPs) within cells, maintaining a persistent pro-inflammatory transcriptional environment, thus facilitating sustained activation of the inflammatory response [60]. Recent studies of cytosolic nucleic acid sensing mechanisms offer new insights into potential interactions between cuproptosis and pyroptosis. Previous research has indicated that cytosolic DNA sensors and their downstream STING signaling pathways can mutually promote inflammasome activation, enhancing the likelihood of pyroptosis under certain conditions [60]. In the context of SCI pathology, copper ion accumulation-induced mitochondrial damage may lead to the release of nucleic acid-based danger signals into the cytoplasm, triggering the STING-related inflammatory pathway [39,63,113]. This process not only may increase the likelihood of pyroptosis but also could intensify its inflammatory amplification effects [39,63,113].

Cuproptosis, ferroptosis, and pyroptosis exhibit converging downstream effects, primarily through their amplifying influence on the inflammatory response and immune cell phenotype remodeling. Cuproptosis often maintains inflammation through mitochondrial metabolic dysfunction and protein homeostasis disruption, releasing immune-stimulating DAMPs that sustain the activation of the inflammatory response [114]. Ferroptosis more directly results in the accumulation of oxidized lipids and their metabolic products, which not only stimulate inflammation but may also affect the cell’s ability to phagocytose and regulate inflammatory signaling, further exacerbating local inflammation [115,116]. In contrast, pyroptosis induces rapid release of a large number of pro-inflammatory cytokines *via* membrane pore formation and cell lysis, with significant peripheral immune cell recruitment, especially during the acute phase [117]. Notably, ferroptosis and inflammation pathways are bidirectionally regulated, and the derivatives of oxidized lipids may also indirectly influence pyroptosis by modulating inflammasome activation [118]. Therefore, enhanced inflammation observed within the same injury site is often not caused by a single form of cell death but rather the combined effect of multiple mechanisms [118].

To more accurately distinguish between different types of cell death mechanisms, studies should prioritize identifying the key features unique to each mechanism. Cuproptosis can be identified based on characteristic molecular changes, such as abnormal aggregation of acylated proteins in the TCA cycle, disruption of iron-sulfur cluster protein homeostasis, and the altered mitochondrial dependence on metabolic status [26]. Ferroptosis can be assessed through the reduction of GPX4 expression, accumulation of polyunsaturated fatty acid phospholipid peroxidation products, and molecular changes related to BSCB damage [110]. Pyroptosis is often accompanied by inflammasome-related caspase activation, cleavage of gasdermin proteins, and the release of interleukin-1β[119]. By assessing the dynamic indicators of metabolic protein homeostasis, lipid peroxidation levels, and inflammasome pore formation in the same SCI stage, and combining immune cell infiltration with transcriptomic expression profiles, it will be possible to more comprehensively reveal the parallel evolution of cuproptosis, ferroptosis, and pyroptosis in the immune microenvironment and their potential synergistic amplification effects [120,121].

## 6. Conclusion

Cuproptosis, a recently discovered form of programmed cell death, offers a new perspective for understanding and treating spinal cord injury (SCI). This mechanism depends on mitochondrial metabolic activity, where an increase in copper load following injury leads to abnormal binding of copper with acylated substrates in the tricarboxylic acid (TCA) cycle. This binding induces protein aggregation, disrupts the stability and function of iron-sulfur cluster proteins, impairs mitochondrial respiration, and causes energy supply deficits. The accumulation of mitochondrial dysfunction may also promote the release of immune-related signals, activate inflammatory transcription programs, exacerbate the imbalance in the function of neurons, glial cells, and immune cells, and drive the progression of secondary tissue damage. Disruption of copper homeostasis not only serves as the trigger for cuproptosis but also sustains oxidative stress and immune activation, continuously disrupting the local microenvironment of the injury site.

Studies have shown that, in SCI and related animal models, molecular changes associated with cuproptosis typically occur alongside an abnormal increase in copper load, and these changes exhibit potential for intervention. For instance, limiting copper accumulation or alleviating copper-induced mitochondrial stress has shown improvements in mitochondrial function markers in animal studies, which correlate with recovery in motor function. However, given copper’s essential role in various metal enzymes, future studies need to further clarify the optimal timing and dosage for intervention, as well as the differential biological effects that may arise from inhibiting local copper activity versus inducing systemic copper deficiency, to enhance the interpretability and clinical translation of the findings.

In clinical research, a combined assessment of copper-related markers in body fluids and cuproptosis-related molecular expressions may support tracking changes in a patient’s condition and provide specific, measurable evaluation criteria for future translational studies. Although this field is still in its early stages, clinical application requires addressing key issues such as delivery specificity and safety assessments, including effective accumulation at the injury site, controlled release rates, and potential impacts on overall copper homeostasis. Optimizing local delivery strategies, establishing a system of efficacy biomarkers, and systematically evaluating long-term safety will lay a solid foundation for translating cuproptosis-related mechanisms from basic research to clinical validation.

Future studies should focus more on the specific manifestations of cuproptosis in different cell types and further explore its relationship with other regulatory forms of cell death. It is necessary to conduct comprehensive evaluations of intracellular copper species, abnormal aggregation of acylated substrates, iron-sulfur protein stability, and mitochondrial function within the same study design, while conducting longitudinal comparisons across different stages of the disease to identify the temporal sequence and causal relationships of these changes. By integrating genetic interventions with cutting-edge technologies like spatial omics and single-cell analysis, researchers may be able to identify the most susceptible cell populations, determine the order of occurrence of cuproptosis in relation to ferroptosis, pyroptosis, and other mechanisms in different cell types, and further reveal their role in regulating the immune microenvironment and limiting tissue repair.

In summary, cuproptosis not only unveils a novel molecular mechanism for secondary injury after SCI but also provides new theoretical foundations and therapeutic strategies for targeting copper homeostasis regulation, maintaining mitochondrial function, and improving the immune microenvironment. With the continuous development of precision medicine and immune intervention techniques, exploring the mechanistic role of cuproptosis and its potential as a therapeutic target is expected to open new ­directions for SCI treatment.

## Data Availability

No datasets were generated or analysed during the current study.
